# Modulating antioxidant systems as a therapeutic approach to retinal degeneration

**DOI:** 10.1016/j.redox.2022.102510

**Published:** 2022-10-17

**Authors:** Xiaoyuan Ren, Thierry Léveillard

**Affiliations:** a Department of Genetics, Sorbonne Université, INSERM, CNRS, Institut de la Vision, 17 rue Moreau, F-75012 Paris, France; b Division of Biochemistry, Department of Medical Biochemistry and Biophysics, Karolinska Institutet, Stockholm, 17177, Sweden

**Keywords:** Retinal Degeneration, Oxidative stress, Antioxidant enzymes, Gene therapy, Rod-derived cone viability factor, Thioredoxin-interacting protein

## Abstract

The human retina is facing a big challenge of reactive oxygen species (ROS) from endogenous and exogenous sources. Excessive ROS can cause damage to DNA, lipids, and proteins, triggering abnormal redox signaling, and ultimately lead to cell death. Thus, oxidative stress has been observed in inherited retinal diseases as a common hallmark. To counteract the detrimental effect of ROS, cells are equipped with various antioxidant defenses. In this review, we will focus on the antioxidant systems in the retina and how they can protect retina from oxidative stress. Both small antioxidants and antioxidant enzymes play a role in ROS removal. Particularly, the thioredoxin and glutaredoxin systems, as the major antioxidant systems in mammalian cells, exert functions in redox signaling regulation via modifying cysteines in proteins. In addition, the thioredoxin-like rod-derived cone viability factor (RdCVFL) and thioredoxin interacting protein (TXNIP) can modulate metabolism in photoreceptors and promote their survival. In conclusion, elevating the antioxidant capacity in retina is a promising therapy to curb the progress of inherited retinal degeneration.

## 1 Introduction

The oxygen constitutes 21% of the atmosphere we live in. However, 3.5 billion years ago when the first life-like structure appeared, the components of the atmosphere were mainly reducing gases. It was until 2.5 billion years ago that oceanic cyanobacteria started producing oxygen and led to the great oxidation event (GOE), which reformed the ecology. Aerobic respiration became favored by organisms due to the much higher energy efficiency that oxidative phosphorylation generates; 38 ATP per glucose, while anaerobic respiration only yields 2 ATP [1].

Although the effort to understand the importance of oxygen for life has been made since the 1600s, it was until the 1770s three scientists made the fundamental breakthrough. The British chemist Joseph Priestley and Swedish chemist, Carl Wilhelm Scheele, discovered the oxygen from independent experiments and named the gas “dephlogisticated air” and “fire-air (*Feuerluft*)”. However, their discovery was shadowed by critics on the phlogiston theory. Inspired by the two chemists, the French scientist Antoine-Laurent Lavoisier proposed the theory of combustion. In 1779, he recognized an essential component in the air for combustion and named it “oxygen (*oxygène*)”. By doing experiments on animals and humans, Lavoisier, for the first time, linked the human metabolic chain to combustion. His scientific contribution led to the fall-down of the phlogiston theory and opened a new era for chemistry and biology [2,3].

As the Hungarian Nobel laureate Albert Szent-Györgyi put it: “Life is nothing but an electron looking for a place to rest”. If we consider life as the flow of energy, the movement of electrons stays in the center of energy. We can even divide all living organisms based on the way of managing electron flow: the photosynthetic organisms utilize sunlight to push the electrons to the top of energy hills; and the rest, including us, live on the energy released by electrons rolling down from higher energy level to lower energy level. Oxygen is an ideal lowland for electrons as each molecule of O_2_ needs four additional electrons to couple all unpaired electrons on its spin orbitals [4]. When O_2_ gets electrons, it is reduced. Similar concept can be seen in redox biology when molecules or proteins receive electrons, they get reduced; when they loss or donate electrons, they get oxidized.

**Fig. 1:**
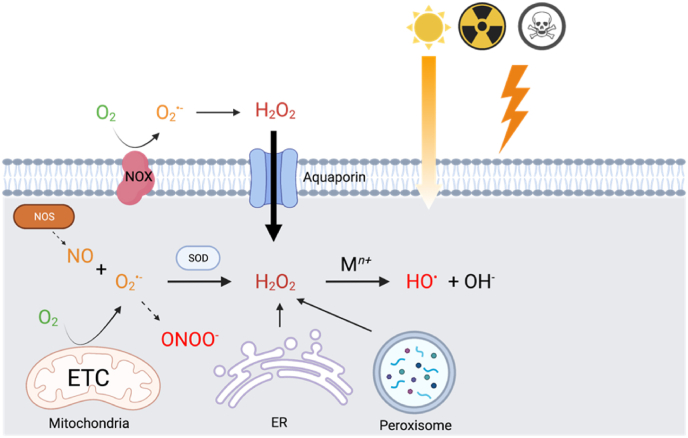
**Production of ROS in cells.** ROS are produced via the leaking of electrons from the mitochondrial respiration chain, or through NOX under physiopathological conditions. ER and peroxisome also contribute to cellular ROS production. NO can be synthesized by NOS. NO and O_2_^·-^ form peroxynitrite. Superoxide dismutases (SODs) can convert O_2_^·-^ into H_2_O_2_ which goes through the Fenton reaction to yield HO^•^.

The utilization of oxygen comes with a price, the production of reactive oxygen species (ROS). More than 95% of the oxygen is reduced by the mitochondrial electron transport chain (ETC) into water by complex IV without the production of ROS. However, when oxygen tension in mitochondria is low near complex IV and can't cater for this reason the need of oxidative phosphorylation, electrons can leak from ETC and be captured by the other molecules, such as oxygen to generate ROS. The iron-sulfur clusters (Fe–S) play an essential role in mitochondria by mediating the electron transfer within ETC, mainly in complex I, II and III, where the electron leakage occurs simultaneously [5]. Oxygen accepts one electron becomes superoxide anion, which sequentially takes another electron and two protons to become hydrogen peroxide (H_2_O_2_). If two electrons were taken, H_2_O_2_ will be decomposed directly into water via dismutation. If one more electron is taken by H_2_O_2,_ it will be split into a hydroxyl anion (OH^−^) and a hydroxyl radical (HO^•^) via Fenton reaction, which is catalyzed by the transition metal (M^*n+*^). Hydroxyl radical is fiercely reactive and can cause damage to DNA, lipids, and proteins. Mitochondria are widely accepted as the major source of endogenous ROS, but ROS can also be produced via endoplasmic reticulum (ER) and peroxisome, where electron transferring happens a lot [6]. However, the saying is challenged by Brown and Borutaige in a review based on the scarcity of quantitative data [7]. The contribution of ROS production depends on cell type and cellular status and the interaction between different cellular compartments. In other studies, it was estimated that 35% of total ROS was formed by peroxisome in rat liver [8] and 25% was generated by ER during oxidative protein folding [9]. Some enzymes produce ROS as their main function to defend the host from invading microbes, such as NADPH oxidases (NOXs), nitric oxide synthases (NOSs) that produce nitric oxide (NO). NO is not a reactive free radical itself, while when superoxide is around, they form peroxynitrite (ONOO^-^), a more oxidizing ROS [10]. Exogenous factors, such as light, radiation, chemicals, also contribute to the production of ROS [11] (Fig. 1).

**Fig. 2:**
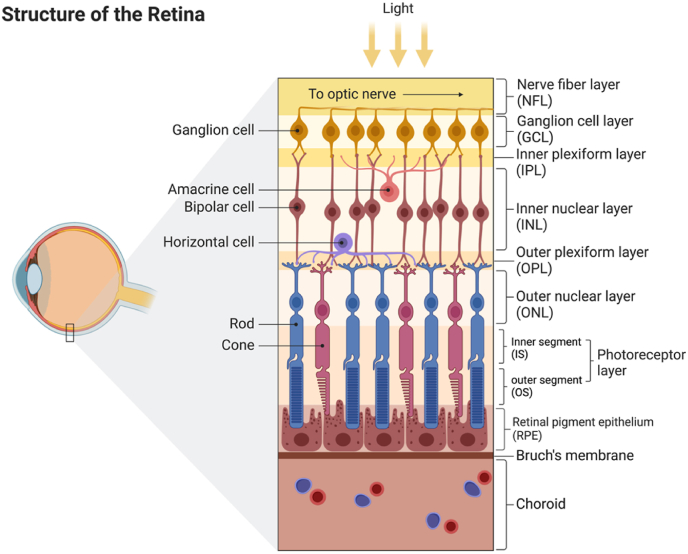
The structure of human retina.

The human retina (Fig. 2), without exception, is facing the challenge of ROS. As a part of the central nervous system (CNS), retina shares the same metabolic property as the brain that a huge amount of energy is needed [12]. There are several evolutionary examples that animals inhabiting in the darkness generally regress their vision systems as an energy-saving strategy [13]. Comparing the Mexican tetra *Astyanax mexicanus* from the surface with an eyeless cave ecotype, the vision energy expenditure was estimated as 15% of the total neural energy cost [14]. Human photoreceptors need ATP to sustain the Na^+^ influx via the cyclic guanosine monophosphate (cGMP)-gated channels and keep depolarized in dark. In response to light, the channels close and energy consumption decreases. However, the decrease of energy usage in cones is much less than in rods when the light is bright. Different from rods, the influx of Na^+^ through cGMP-gated channels doesn't drop further, while the rods are hyperpolarized and saturated. Thus, cones are responsible for daylight vision but consume more energy in bright light [15]. The feature may also explain why rods outnumber cones in duplex retina of mammals from the perspective of energy saving [16]. Cones contain more mitochondria than rods and generate more endogenous ROS via energy production, making them more susceptible to oxidative stress [17].

In addition, retina is constantly exposed to sunlight and artificial light that trigger the production of ROS, especially in the presence of photosensitizers such as retinoids [18], and leads to photooxidation [19]. Especially during the progressive loss of photoreceptors in retinal degeneration, oxygen supply from choroidal circulation cannot adjust accordingly and the remaining photoreceptors are exposed to a higher level of oxygen [20].

As stated above, excessive ROS can cause damage to biological molecules, such as DNA, lipids, and proteins. ROS can modify the purine and pyrimidine bases, the deoxyribose backbone, and result in DNA break or abnormal crosslink. Both nuclear and mitochondrial DNA can be targeted by ROS that can lead to mutagenesis and mitochondrial dysfunction. When the DNA repair machinery fails, apoptosis will be initiated. In the *rd1* mouse, an animal model of hereditary retinal degeneration, the DNA damage marker 8-hydroxy deoxyguanosine (8-OHdG) and proliferating cell nuclear antigen (PCNA), a marker of DNA repair, can be found in the outer nuclear layer (ONL) [21,22].

Retina is especially vulnerable to ROS because photoreceptors are rich in polyunsaturated fatty acids that are susceptible to lipid peroxidation [23]. Peroxidation of membrane lipids will disrupt the integrity of cellular membranes and alter the membrane dynamic and permeability, finally induce cell death. In addition, lipid peroxides can also participate in the Fenton reaction to generate extremely reactive hydroxyl radicals. A recently emerged type of cell death, ferroptosis, is found to be triggered by lipid peroxidation [24]. In *rd1* mouse, acrolein, a product of lipid peroxidation, increased significantly upon the death of photoreceptors [25]. Increased level of thiobarbituric acid reactive substances (TBAR), another marker of lipid peroxidation, was detected in *rd10* photoreceptors [26,27].

Proteins can be damaged by ROS in different ways. Firstly, ROS can oxidize key cysteines and lead to loss of functions of the proteins. Some of the oxidation are reversible, while some are irreversible and permanently inactivate the proteins, for example, sulfonation [28]. Secondly, iron-sulfur cluster proteins, such as mitochondrial aconitase and complexes (I, II, and III), are redox-sensitive and release iron after being damaged by ROS. The free iron will take part in the Fenton reaction and exacerbate oxidative stress [29]. Thirdly, excessive ROS interfere with protein folding and provoke ER stress that leads to cell death [30].

Indeed, the accumulation of oxidative stress has been recognized as a hallmark of retinal degeneration. Retinitis pigmentosa (RP) is a retinal degenerative disease, caused by mutations in at least 69 genes, characterized by progressive death of photoreceptors, rods then cones [31,32]. Increased oxidative stress markers and decreased antioxidant capacity were observed in animal models of RP [33] and aqueous humor samples from RP patients [34,35]. Chronic inflammation mediated by macrophages or glial cells is often observed in retinal degeneration. Constant production of NO by NOSs and ROS by NOXs contributes to the damage [36]. Inhibition of NOS or NOX reduces the production of ROS and significantly rescued cone cell death in *rd1* RP model [37,38]. The rod specific cGMP phosphodiesterase (PDE6) is crucial in phototransduction by hydrolyzing cGMP and sequentially leading to the closure of cGMP-gated ion channels. *PDE6B,* encoding the beta-subunit of PDE6, was one of the first genes recognized causing recessive RP. Non-sense mutation of *Pde6b* in *rd1* mice resulted in accumulation of cGMP leading to increased influx of Ca^2+^. The prolonged opening of ion channels consumes more energy, therefore leads to metabolic overload to increase ROS and cause toxicity and death of rod-photoreceptors [39].

Age-related macular degeneration (AMD) is one the leading causes of vision loss that affects millions of the elderly. Both aging and chronic inflammation play a central role in the pathogenesis of AMD [40]. Increased oxidative stress is often observed in age-related diseases. AMD patients exhibited a higher level of oxidative stress both in serum and in primary RPE cells from AMD donors, suggesting both systemic and local oxidative damage [41,42]. Epidemiology studies have found a strong link between cigarette smoking and AMD. Cigarette smoke is a rich source of oxidants that can cause cellular damage [43].

Mammalian cells, as other eukaryotic cells, are equipped with antioxidant systems to counteract ROS and therefore maintain a redox homeostasis, which is steady balance between ROS production and removal to keep the normal physiological functions [44]. During the last few decades, our understanding about ROS has evolved; ROS can act as signal molecules to mediate signaling rather than just act as a residue of damaging molecules. For example, H_2_O_2_ has recently been recognized as an important redox signaling molecule involved in various biological processes [45]. Lipid peroxides, as the downstream products of H_2_O_2_, were found to mediate retinal development and regulate retinal progenitor cell differentiation in zebra fish. During the development, the expression of catalase (CAT), one of the major enzyme to decompose H_2_O_2_, was fine tuned to maintain a redox homeostasis via the modulation of lipid peroxidation [46]. A large part of signal transduction is via the oxidation of cysteine residues within proteins. Cysteines are susceptible to ROS modification and most, but not all, of the modifications can be reverted by the antioxidant systems. Thus, cysteine modification can serve as a redox signaling switch with some similarities with protein phosphorylation. Therefore, the antioxidant systems not only defend cells from the insult of ROS but also regulate redox signaling. We will focus now on the role of the antioxidant systems in the retina and explore their therapeutic potential to treat retinal diseases.

## 2 Antioxidant systems

### 2.1 Small antioxidant molecules

Small antioxidant molecules can directly react with ROS non-enzymatically by donating or accepting electrons to eliminate the unpaired status of the radicals. Some of them can be taken up exogenously. Ascorbate (Vitamin C), for example, is a water-soluble antioxidant with multiple biological functions. The concentration of ascorbate in the retina of guinea pigs is much higher (∼20 mg/dl) compared to plasma (less than 1 mg/dl), and most of it is in reduced form [47]. Using radioactive ascorbate, Hosoya's group found that ascorbate is mainly transported across the blood-retinal-barrier (BRB) as its oxidized form, dehydroascorbic acid (DHA), through facilitative glucose transporters, GLUT1/3 [48]. Since glucose metabolism is at the origin of ROS production by the mitochondria, the import to photoreceptors of DHA by GLUT is rational. Most of the biological function of ascorbate depends on its reduced form and the concentration of DHA in cells is low. Uptaken DHA can be reduced to ascorbate rapidly in cells by the readily existing systems, such as glutathione (GSH) and other reductases [49]. After light exposure, the ratio of ascorbate/DHA drops and DHA was predominantly found in pigment epithelium-choroid complex in guinea pigs [47].

GSH is the most abundant low molecular thiol containing molecules in eukaryote cells with an estimated concentration at the millimolar level. GSH not only directly reacts with ROS and electrophiles, but also donates electrons to GSH-dependent antioxidant enzymes, such as GSH peroxidases (GPXs) and GSH transferases (GSTs). GSH is synthesized in cells in two steps. The first step is to link a cysteine and a glutamate to form γ-glutamylcysteine which is carried out by the glutamate cysteine ligase (GCL), that is a heterodimeric protein composed of catalytic (GCLC) and modifier (GCLM) subunits. In the second step, catalyzed by GSH synthetase, a glycine is added to the γ-glutamylcysteine. The first step is considered as the rate-limiting step in GSH synthesis [50]. More than 98% of the cellular GSH is in its reduced form. After reacting with ROS or donating electrons, GSH can be oxidized in GSSG. GSSG can be reduced back to GSH by glutathione reductase (GR), which utilizes NADPH as the electron donor. The ratio between GSH/GSSG is often used as an indicator of cellular redox status, highest value of this ratio corresponds to the highest reducing power [51]. The presence of GSH in retina cells has been well-documented and a significant reduction of the GSH/GSSG ratio has been found in aqueous humor from RP patients [52]. Interestingly, in the *rd1* and *rd10* mouse model of RP, GSH/GSSG ratio decreased significantly when rod-photoreceptors started dying, while the catalytic subunit of GCL, GCLC, increased at the protein level, probably trying to compensate the increased level of ROS [53]. Surprisingly, using immunochemical method, there were studies detected little GSH in outer segments of rods and cones. Based on the observations, Winkler hypothesized that the renewal of outer segments is a strategy to cope with the lack of the important molecules that renewal serves as a “surrogate antioxidant” [54].

Vitamin E (α-tocopherols) is a family of lipid-soluble antioxidants with eight isoforms that protects polyunsaturated fatty acids from oxidative damage by superoxide, hydroxyl radical and peroxides. During the reaction, vitamin E can transfer the phenolic hydrogen atom to oxidants to quench the reactivity of ROS. The resulting tocopherol radical can be regenerated by ascorbate [55]. Several animal models have shown that vitamin E deficiency can lead to retinal degeneration, characterized by the disruption of photoreceptor outer segment membranes, and aggravated the death of retinal ganglion cell death in a rat model of glaucoma [[56], [57], [58]].

Carotenoids are a group of lipid-soluble pigments well-known as vitamin A precursors. Humans absorb carotenoids from diet efficiently and accumulate them in adipose and other tissues. Photoreceptors utilize a photosensitive visual chromophore to absorb light and initiate the phototransduction, 11-*cis*-retinal, which is converted from vitamin A (all-*trans*-retinol). The 11-*cis*-retinal can bind covalently to opsins to form visual pigments that can be activated by light and release all-*trans*-retinal, which can be reduced back to vitamin A. The process is called visual cycle [59]. In human retina, the concentration of carotenoids can reach a level between 0.1 and 1 mM in the central fovea, 1000 times higher than in other tissues, suggesting their important roles in visual functions [60]. Interestingly, among all the carotenoids, only two of them are specifically distributed in retina, especially in macula, namely lutein and zeaxanthin [61]. The lutein and zeaxanthin can protect photoreceptors in two ways. First, they localize mostly in the outer plexiform layer and form a filter for blue light, the high-energy short-wave light that can penetrate the cornea and lens to reach the retina directly and induce photochemical damage [62]. Secondly, lutein and zeaxanthin can quench singlet oxygen (^1^O_2_) to its ground state and protect photoreceptors from light-induced lipid peroxidation [63].

Melatonin is well-recognized for its role in regulating circadian rhythms. Circulating melatonin in humans is primarily synthesized in the pineal gland but local synthesis of melatonin can occur in several peripheral organs. In the retina, melatonin is produced by photoreceptors because the mRNA of enzymes for melatonin synthesis, such as hydroxyindole-O-methyltransferase (HIOMT) [64] and aralkylamine N-acetyltransferase (AANAT) [65,66] is selectively detected in photoreceptors. Besides its role in circadian regulation, melatonin can direct react with a broad spectrum of ROS, such as hydrogen peroxide, hydroxyl radical (HO^•^), nitric oxide, peroxynitrite (ONOO^-^), and so on and protect cells from oxidative damage [[67], [68], [69]]. Apart from the direct reaction with ROS, melatonin participates in antioxidant defense at multiple levels. It can maintain GSH homeostasis by increasing the GSH content and decreasing the oxidized GSH (GSSG) in brain and liver mitochondria. The activity of GSH-dependent antioxidant enzymes, such as GSH-dependent peroxidase (GPXs) and GSH reductase (GR), was also significantly increased by melatonin. The increase of the GSH-dependent enzyme activity was much higher than GSH, suggesting an upregulation in protein level [70]. What's more, melatonin has the capacity to upregulate several antioxidant enzymes via modulating the Nuclear factor (erythroid-derived 2)-like 2 (NRF2) transcriptional pathway [71], at the same time downregulating prooxidant enzymes that increase the production of ROS, such as lipoxygenases and NOSs [72].

## 3 Thioredoxin and glutaredoxin systems

**Fig. 3:**
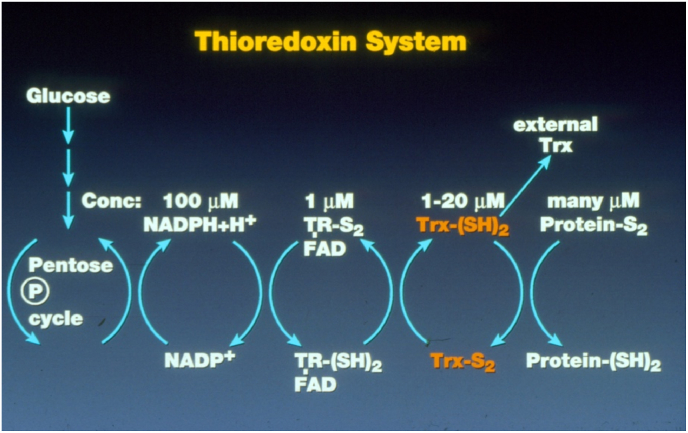
**The thioredoxin system illustrated by Prof. Arne Holmgren.** TR: thioredoxin reductase (TXNRD), FAD: flavin adenine dinucleotide, Trx: thioredoxin (TXN).

**Fig. 4:**
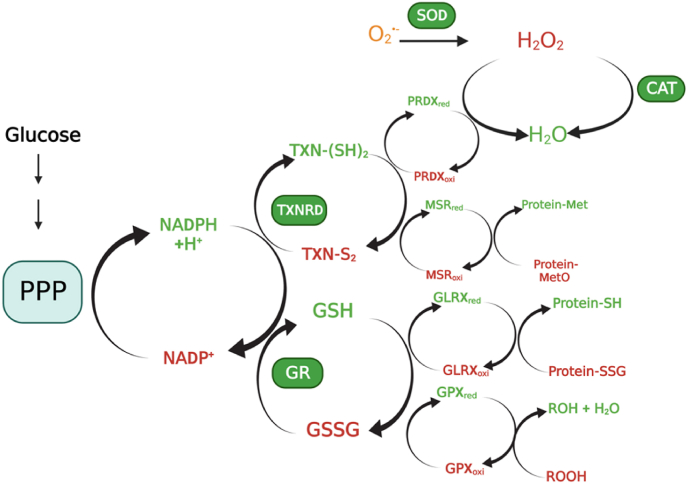
**The antioxidant enzymes in retinal cells.** SOD catalyzes the dismutation of superoxide to hydrogen peroxide. Catalase decomposes H_2_O_2_ to H_2_O. Both TXN and GLRX systems use NADPH from the metabolism of glucose by the pentose phosphate pathway, as the ultimate electron donor to reduce the downstream proteins.

The thioredoxin (TXN) and glutaredoxin (GLRX) systems are the two antioxidant systems evolutionarily conserved from bacteria to humans. The TXN system comprises NADHP, TXN reductase (TXNRD), and TXN (Fig. 3), the GLRX system, NADPH, GSH reductase (GSR), GSH, and GLRX. Both systems use NADPH as the ultimate electron donor. For the TXN system, TXNRD receives electrons from NADPH to reduce the oxidized TXN. For the GLRX system, GSR uses NADPH to reduce GSSG to GSH that reduces oxidized GLRX. TXN and GLRX, as the antioxidant executors, not only counteract oxidative stress but also take part in various biological signaling processes in cells by interacting with their downstream targets. (Fig. 4).

### 3.1 TXN

TXN was originally identified in *Escherichia coli* as the electron donor for ribonucleotide reductase (RNR), the enzyme catalyzing the formation of deoxyribonucleotide *de novo* for DNA replication and repair. Holmgren and coworkers first solved the structure of *E.coli* TXN in 1975 what revealed that the protein is built up by a central core of 5 β strands surrounded by four α helices with a signature active site sequence -Cys-Gly-Pro-Cys- (-CGPC-) which is located at the end of a β strand (β2) and the beginning of a long α helix [73]. Later, more proteins were found to have a similar structure, known as the thioredoxin-fold, and grouped into the TXN superfamily. There are three canonical TXN paralogs widely distributed in human tissues, the cytosolic TXN1 (encoded by *TXN*), the mitochondrial TXN2 (encoded by *TXN2*), and a testis-specific TXN (encoded by *NME9*). Typically, TXNs perform thiol-disulfide exchange reactions via the catalytic active site cysteine pair, the -CXXC- motif. Each catalytic cycle results in the formation of a disulfide between the cysteine pair due to oxidation.

The first study about TXN in the retina was done by Hansson and Holmgren who checked the change of insulin-like growth factor 1 (IGF1) expression during the development of retina in rat. Since IGF1 is a known substrate of TXN, the distribution of TXN, TXNRD and RNR was checked [74]. Immunoreactivity showed that TXN and TXNRD were equally expressed by the nerve cells in the inner nuclear layer (INL), retinal ganglion cells (RGCs) and photoreceptors throughout the development of retina. While, photoreceptors showed a reduction of TXN and TXNRD with retinal maturity [74]. RNR, the primary target of TXN, was observed in the first few postnatal days (PND) in the INL and photoreceptors, then it vanished from PND12 and only expressed in scattered endothelial cells. Early studies often reported colocalization of the TXN system and RNR, but not in the matured retina, suggesting the TXN system carries out other functions than supplying electrons to RNR that would primarily be involved in DNA repair [74]. This observation correlates with the exit of the cell cycles of retinal precursors during their maturation [75].

Oxidative stress markers, such as lipid peroxides and peroxynitrite, have been seen in retinal ischemia-reperfusion injury [76]. Ischemia-reperfusion is a paradoxical tissue response that is manifested by blood flow-deprived and oxygen-starved organs following the restoration of blood flow and tissue oxygenation [77]. Ohira and colleagues hypothesized the antioxidant role of TXN in retina and found that TXN was induced in RPE in ischemic retina after reperfusion, and also by light exposure injury [78]. The expression of TXN was also induced in mitochondria in H_2_O_2_ treated RPE cells [79]. One should notice that it was before the discovery of mitochondrial TXN2, and these observations may actually by attributable to TXN2. These studies highlighted the role of TXNs in defending RPE cells against ROS. Prostaglandin E1 (PGE1) is a commonly used for treating obstructive diseases, including ischemic retinopathy. Using a PGE1 analog, the same group found that the treatment preserved TXN in retina up to 14 days in a retinal ischemia/reperfusion rat model, while TXN diminished in the non-treatment group. This study suggests that TXN may suppress oxidative damage thus contribute to the therapeutic effects of PGE1 [80].

TXN exhibited a protective activity against retinal photic injury in mice, as well. After light exposure, TXN was induced in the INL and ONL of the retina as an antioxidant response to oxidative stress. Intravitreous administration of TXN1 or transgenic overexpression of TXN1 preserved a significantly number of photoreceptors. The protection could not be achieved by using TXN1 with active site cysteine mutations (C32S/C35S) [81,82]. This is consistent with the fact that the active site cysteine pair is essential for TXN's enzymatic activity.

Glutamate is the principal excitatory neurotransmitter that stimulates *N*-methyl-d-aspartate (NMDA) receptors in the central nervous system. Nevertheless, glutamatergic excitotoxicity is causing damage to neurons. Several neurons in the retina express NMDA-receptors, such as RGCs and amacrine cells [83]. NMDA administration has been used to establish retinal degeneration models [84]. In a rat model of NMDA-induced retinal damage, intravitreal injection of TXN1, but not the active site mutant (C32S/C35S) protein, protect RGCs from NMDA-induced apoptosis by inhibiting c-Jun N-terminal kinase and the mitogen-activated protein kinase (MAPK) p38 pathways and by reducing oxidative stress [85].

Not only TXN1, the mitochondrial TXN2, contributes to the protection of RGCs after optic nerve transection, oxidative stress, and experimental glaucoma [[86], [87], [88], [89]]. Some studies show an even better effect of TXN2 on promoting RPE survival than TXN1 (see above). The difference may be due to the ability of TXN2 to maintain the mitochondrial membrane potential [90]. TXN2 is also upregulated after human umbilical cord blood mesenchymal stem cells administration by intravenous or intravitreal routes on the cryo-induced retinal injury model [91].

Some compounds and treatments protect retina from light-induced damage and the cytoprotective effects were attributed to the induction of TXN1 [[92], [93], [94], [95], [96], [97], [98], [99], [100], [101]] or TXNRD1 [102]. Cao's group found that the expression of TXN1 and TXNRD1 was impaired in *tubby* mice, a retinal degeneration model [103]. Treating *tubby* mice with sulforaphane increased the level of TXN1 and TXNRD1 at both mRNA and protein level via the NRF2 antioxidant signaling pathway. The same mechanism can explain the adaptive upregulation of TXN1 and TXNRD1 induced by light exposure [104]. Later, the same group showed transgenically expressed TXN1 driven by beta-actin promoter, expressed in the inner segment, outer plexiform layer (OPL), inner plexiform layer (IPL), and ganglion cell layer (GCL), provides both functional and morphological protection to photoreceptors of the *tubby* mice. By exploring the mechanism, the authors found that TXN1 upregulated mRNA and protein level of neurotrophic factors brain-derived neurotrophic factor (BDNF) and glial cell line-derived neurotrophic factor (GDNF), and activated survival signaling pathways such as AKT/RAS/RAF1/ERK, while inhibited the ASK1/JNK apoptosis pathway [105].

### 3.2 TXN targeted proteins

#### 3.2.1 Peroxiredoxins (PRDXs)

Peroxiredoxins (PRDXs) are ubiquitous enzymes catalyzing the neutralization of H_2_O_2_, lipid peroxides, and peroxynitrite. To date, 6 mammalian PRDX paralogues have been discovered, all are expressed in rat and marmoset retina and optic nerve [106] (Table, 1). All paralogues contain the N-terminal cysteine which can be oxidized by their chemical substrates rapidly and selectively during catalysis [107]. In most cases, the N-terminal cysteine (the peroxidatic cysteine) is oxidized by substrates into a sulfenic acid (-SOH), then a second cysteine at C-terminus (the resolving cysteine) reduces the sulfenic acid and forms an intermolecular (PRDXs 1–4) or intramolecular (PRDX5) disulfide which can be reduced by TXNs [108]. PRDX6, the 1-Cys PRDX, lacks the resolving cysteine, therefore, it does not form disulfide and cannot be reduced by TXN. Instead, the oxidized cysteine in PRDX6 is reduced by GSH catalyzed S-transferase isoform π (GSTP1) [109]. PRDXs are efficient peroxidases with an estimated rate constant (*k*_*cat*_) of 10^7^–10^8^ M^−1^ s^−1^ [110]. Paradoxically, a study found that human PRDX1 can be inactivated by as little as 100 μM H_2_O_2_ and this inactivation was shown to be due to the hyperoxidation of the N-terminal cysteine into a sulfinic acid (–SO_2_H), which cannot be regenerated by TXN, as stated [111]. This inactivation is called the “floodgate model”, which is important for redox signaling since it allows non-stress H_2_O_2_ produced by NOX to get stabilized and exert its signaling function [107]. One example is the study done by Reddy and Millar's group showing that the oxidation and reduction of PRDX is a transcription-independent rhythmic cycle conserved during evolution, the rhythmic oxidation of PRDX persisted even in constant darkness or in the presence of transcriptional inhibitor, the characteristic of a circadian oscillator. Therefore, PRDX oxidation serves as a post-translational circadian biomarker shared between human red blood cells and the ancestral unicellular species of marine green alga [112].

PRDX6 was firstly checked in bovine eyes and they were found in the outer plexiform layer, blood vessel of the retina, and in choroidal blood vessel [113]. In the primate retina, PRDX3 was found mainly in the photoreceptor inner segments, especially the cones sensing blue light in the macaque retina, the outer and IPL, and the RGCs [114]. A proteomic profiling study comparing retinal and choroidal endothelial cells showed that PRDX4 is one of the 11 proteins most abundant in retina, suggesting its importance for vision [115]. PRDX2 was found abundantly expressed in rat retina including all layers from the GCL to the retinal pigment epithelium (RPE); the most intense labeling was recorded for ganglion cells. In the *rd1* mouse, a model of recessive RP, low dose irradiation delayed neurodegeneration by upregulating PRDX2 protein expression [116]. The induction of *Prdx2* mRNA was due to the antioxidant NRF2 transcriptional pathway [117]. PRDX1/2/6 were upregulated in retina under various oxidative stress conditions [[117], [118], [119], [120]] as a cellular protective mechanism [[121], [122], [123], [124]]. PRDX3/4/5/6 were observed in photoreceptors, RPE, and choroids [125]. PRDX6 could be detected throughout the whole Müller cell body and serves as a novel Müller cell marker in the mouse retina [120]. Overexpressing PRDX6 rendered Müller cells resistant to oxidative stress [126]. Interestingly, Akagi's group reported that *Prdx5* and *Prdx6* were the most expressed *Prdx* genes in rat retina among the 6 paralogues and overexpressing them protected pig retinal pericytes from high glucose-induced oxidative damage [127]. PRDX6 was also significantly induced in porcine retinal homogenate after glutamate treatment, suggesting a protective role in retina against excitotoxicity [128]. Overexpression of PRDX6 attenuated TNF-alpha and glutamate-induced RGC death not only by limiting ROS levels, but also curtailing NF-kappa B activity, and maintaining Ca^2+^ homeostasis [129,130]. In a mouse model of Leber congenital amaurosis (LCA) after gene therapy with AAV-delivered RPE65, a proteomic study found PRDX6 was significantly upregulated among 39 other proteins.

#### 3.2.2 Methionine sulfoxide reductases (MSRs)

Methionine, as another sulfur-containing amino acid, is susceptible to oxidation by ROS to methionine sulfoxide (MetO). Similar to cysteine oxidation, the formation of methionine sulfoxide in proteins can lead to altered protein structures or functions. Methionine sulfoxide reductases (MSRs) catalyze the reduction of methionine sulfoxide to methionine. Due to the asymmetric nature of the sulfur atom in the side chain of methionine, two diastereomers of sulfoxide can be produced, methionine-S-sulfoxide (Met-S-SO) and methionine-R-sulfoxide (Met-R-SO), that can be repaired by MSRA and MSRB1-3, respectively [131]. MSRA isoforms can be found in both cytosol and mitochondria. MSRB1 is cytosolic, MSRB2 is a mitochondrial protein, while MSRB3 is located in both mitochondria and ER [132]. It should be noticed that MSRB1 is a selenoprotein with a selenocysteine (Sec), which renders it more efficient to reduce Met-R-SO compared to its cysteine version [133]. Despite different catalytic mechanisms, all MSRs receive electrons from the TXN system [134].

In the early stage of discovering MSRs, CBS-1, later named MSRB2, was found expressed in retina at the highest level among other tissues [135]. In the monkey retina, MSRB2 is localized in the GCL, the OPL, and the RPE. MSRB2 is most pronounced in the OPL of the macula and foveal regions suggesting an association with the cone mitochondria. Overexpression of MSRB2 in the RPE rendered them resistant to oxidative stress [136]. Actin dysregulation has been found during the pathogenesis of many inherited retinal degenerative diseases [137]. The semaphorin/plexin signaling is essential for actin filament (F-actin) dynamics. Mutations of a semaphorin-encoded gene, SEMA4A was identified in a group of RP patients and cone-rod dystrophy (CRD) [138]. Molecule Interacting with CasL (MICAL), an important mediator of semaphorin/plexin signaling, destabilize F-actin by oxidizing the key methionines in F-actin to Met-R-SO [139]. MSRB1 has been recognized as the key enzyme to reverse the methionine oxidation of F-actin, and take part in the regulation of the actin assembly [140,141].

MSRA was also found throughout the rhesus monkey (*Macacca mulatta*) retina, especially abundant at the photoreceptor synapses, RGC and Müller cells. Silencing MSRA in RPE made them more sensitive to oxidative stress [142]. The expression of MSRA is driven by two 5’ distinct promoters and the downstream one was found very active in RPE cells. It makes RPE cells have 4.5 folds level of MSRA than the neural retina [143]. MSRA can facility ATP synthesis in mitochondria and increase the phagocytic activity of RPE cells. The effect was independent of its antioxidant ability [144]. The *MsrA* knock-out (KO) mouse model was consistent with RPE expression, but electroretinography (ERG) revealed decreased light responses specifically of cone photoreceptors, suggesting a therapeutic potential in treating cone defects [145].

### 3.3 Glutaredoxin (GLRX)

Glutaredoxin (GLRX) was first discovered by Arne Holmgren in 1976 as a reductase for RNR in a mutant *E.coli* lacking Txn [146]. GLRXs exist in most living organisms from prokaryotes, plants, viruses to eukaryotes. They typically have a TXN fold with a -CXXC- or -CXXS- motif at the active site. To date, four paralogues of GLRXs have been found in humans. They are divided into two groups based on the cysteine numbers at the active site: the dithiol GLRXs including GLRX1 and GLRX2 with a -CXXC- active site motif; the monothiol GLRXs including GLRX3 and GLRX5 with a -CXXS- active site motif [147].

GLRXs are versatile proteins not only reduce disulfide and protein glutathionylation but also play an important role in iron metabolism by coordinating iron-sulfur clusters ([Fe–S]) [148]. When Gladyshev's group firstly identified human GLRX2, they found that the mitochondrial GLRX exhibited only 36% sequence identity with the previously characterized mammalian cytosolic glutaredoxin, GLRX1 [149]. Human GLRX1 contains a -CPYC- active site motif, while GLRX2 possesses a -CSYC- motif. The subtle difference is essential for GLRX2 serving as a [Fe–S] binding protein. When the -CPYC- in human GLRX1 was replaced by -CSYS-, the GLRX2 active site, GLRX1 became a [Fe–S] binding protein, too [150]. The expression and enzymatic activity of GLRXs were found in all ocular tissues except the vitreous body [151,152]. GLRX1 was observed in all layers of retina, most intense immune-staining was detected in GCL. The highest level of GLRX2 was found in the ciliary body and the whole retina, especially in the GCL, IPL and OPL. GLRX3 was consistently labeled the INL and the GCL, while only weak staining was seen in the OPL, INL and ONL. Neither GLRX2 nor GLRX3 was detected in photoreceptors [125]. However, the technical limitation of immunostaining must be kept in mind.

Wu's group reported overexpression of GLRX1 protected RPE cells from H_2_O_2_-induced cell death. They also found GLRX1 attenuated H_2_O_2_-induced protein glutathionylation. Protein kinase B (AKT) is a serine/threonine kinase and the phosphorylation of AKT promotes cell proliferation and survival. GLRX1 stimulates the phosphorylation of AKT by catalyzing its deglutathionylation [153]. GLRX1 was found to be steadily expressed in the cone outer segments in the advanced age retina, suggesting its potential role in protecting cones from aging-related stress [154].

GLRXs are [Fe–S] coordinating proteins that are important for iron metabolism. Iron homeostasis is disturbed in retinal degeneration [155]. Iron overload is related to a newly discovered programmed cell death, ferroptosis, which has been linked to retinal degeneration recently. Ferroptosis can be triggered by the accumulation of peroxides and iron at the same time to start the Fenton reaction. The highly reactive hydroxyl radical produced by the Fenton reaction will disrupt the cell membrane rapidly and initiate the cell death process. The ferroptosis inhibitor, ferrostatin-1 (Fer-1), rescued lipid peroxides-induced RPE cell death and light-induced photoreceptor death, more effectively than apoptosis or necroptosis inhibitors [156,157]. As an iron chelator, deferoxamine (DFO) protected both RPE and photoreceptors from chemical and light-retinal degeneration. GLRXs may curb ferroptosis by coordinating the iron-sulfur clusters to restrain the cellular iron load.

### 3.4 Glutathione peroxidases (GPXs)

GPXs are a group of GSH-dependent peroxidases that catalyze the reduction of peroxides, including H_2_O_2_ and organic hydroperoxides, such as lipid peroxides. There are eight distinct GPXs identified in humans, GPX1-8, among which are five selenoproteins, GPX 1–4 and GPX6 [158]. The rate of seleno-GPXs reacting with H_2_O_2_ is similar to PRXs, so both are considered the main H_2_O_2_ scavenger in mammalian cells and protect them from oxidative stress-induced cell death [159], especially ferroptosis which is recognized as a new mechanism for retinal degeneration [160,161].

Lower total GPXs activity and a higher level of lipid peroxidation were seen in the retina of r*d1* mouse [162]. GPX4, the most studied GPXs in human, has three splicing variants localized in cytosol, mitochondria, and nucleus [163]. Knocking out GPX4 in mice resulted in embryonic death, and highlighted its biological importance [164]. GPX4 is abundantly expressed in the retina, especially in the inner segments (IS) of photoreceptor cells, but also found in RPE and choroid. Deletion of GPX4 in photoreceptors caused lipid peroxidation and drastic cell death at the early stage of retinal development. The results indicate GPX4 is an essential antioxidant enzyme for the maturation and survival of photoreceptors [165]. Overexpression of GPX1 or GPX4 in RPE cells protected them from oxidative stress. Increased expression of GPX4 in mouse photoreceptors reduced paraquat and hypoxia-induced thinning of ONL [166].

### 3.5 Redox signaling mediated by TXNs and GLRXs in retina

As we mentioned, the role of ROS as signaling molecules has been more and more noticed in physiological and pathological conditions mainly via the modification of cysteine in proteins. Thiols (-SH group) in cysteine are susceptible to ROS modification and most of the modifications are reversible. Thus, cysteine modification can serve as a redox signaling switch similarly to protein phosphorylation the main difference being the exchange of the redox status of the protein that is reduced and the reductase that then become oxidized. Reversible redox modification of cysteine includes S-sulfenylation (sulfenic acid, –SOH), sulfinic acid (-SO_2_H), disulfide bond (-S-S-), S-glutathionylation (-SSG), S-nitrosylation (-SNO). If sulfinic acid is further oxidized, it forms sulfonic acid (-SO_3_H), which is irreversible and leads the permanent protein inactivation.

TXNs and GLRXs can regulate redox signaling in different ways. First, they neutralize ROS directly before proteins get modified. Secondly, they can bind to certain transcriptional factors and modulate their activity. Thirdly, they catalyze the reversible redox post-translational modifications to control the redox switch.

TXNs can supply electrons to PRDXs, that convert H_2_O_2_ to H_2_O (Fig. 4), or bind to other proteins to modulate redox signaling. The apoptosis-regulating kinase 1 (ASK1) is an example. ASK1 is a MAP3K (mitogen-activated protein kinase kinase kinases, also known as MAP3K5) activating MAP2K-JNK/p38 signaling cascades which are essential for initiating the ER stress response and cytokine-induced apoptosis. TXN1 was found as a physiological inhibitor of ASK1 by direct protein-protein interaction [167]. Activation of ASK1 has been reported in various mouse models of ocular diseases, including glaucoma, retinal ischemic injury. TXN1 binds ASK1 via disulfide formation between its active site C^32^ or C^35^ and C^250^ in the N-terminal domain of ASK1 and inhibits its kinase activity [168]. TXN system has been considered as the primary system to reduce protein S-nitrosylation by nitric oxide, which is often produced during inflammation [11]. *N*-methyl-*N*-nitrosourea (MNU) is used to induce outer retinal degeneration in mice. MNU treatment led to increased protein S-nitrosylation level that is often seen in inflammation and light exposure [169,170]. TXNs can reduce the reversible redox modification and bring back protein functions [171].

The role of GLRX-mediated redox signaling in retina is still controversial. Mieyal's group observed that GLRX activity increased in the diabetic retina of mice and retinal Müller cells cultured in high glucose medium. Overexpression of GLRX1 in Müller cells is associated with an increased NF-kappa B activity [172]. A later study from the same group found IL6 level was increased in conditioned medium from Müller cells overexpressing GLRX1, which formed a vicious circle to increase further the NF-kappa B and GLRX1 activity. Thus, GLRX1 regulated the proinflammatory responses of Müller cells in both autocrine and paracrine ways. This mechanism may be due to the activity of GLRX that deglutathionylates IκB kinase (IKK), the negative regulator of NF-kappa B [173].

## 4 Modulating antioxidant systems to treat inherited retinal diseases

Regardless of the genetic variation in inherited retinal diseases, oxidative stress commonly contributes to pathogenesis [131]. The cellular damage induced by oxidative stress further triggers inflammation and cell death pathways that exacerbate oxidative stress, making ROS a key chain in this vicious circle [174]. Thus, modulating antioxidant systems to counteract ROS becomes a promising treatment for inherited retinal diseases, at least to slow down the disease progress by breaking the vicious circle.

### 4.1 Small antioxidants

Supplementation of small antioxidants has been tested in several clinical trials for treating RP, including vitamin A &E, docosahexaenoic acid, beta-carotene, lutein. Some of the studies showed a protective effect on patients by reducing the visual field loss and slowing the decline of ERG performance [175]. Although impaired docosahexaenoic acid synthesis was observed in X-linked RP patients, a 4-year-duration randomized controlled trial concluded that long-term docosahexaenoic acid supplementation was not effective in slowing the loss of rod or cone ERG function associated with X-linked RP [176]. It should be noticed that the number of patients enrolled in the trial was small and may not provide enough statistical power. In the *rd1* mouse model of RP, the combination of lutein, zeaxanthin, lipoic acid, and reduced GSH significantly decreased the oxidative damage and rescued photoreceptors, while these antioxidants individually have no significant rescue effect [21].

Small antioxidants supplementation also showed promising therapeutic potential in AMD patients. The Age-Related Eye Disease Study (AREDS) is a long-term prospective study, started in 1992 and 4757 participants were enrolled, designed to evaluate the effects of oral supplements of antioxidants on the progression to advanced AMD [177]. The multi-vitamin and mineral formulation used in AREDS included vitamin C, vitamin E, β-carotene, zinc oxide (ZnO), and cupric oxide (CuO). After five years follow-up, the AREDS formulation significantly slowed down the progression to advanced AMD [178].

### 4.2 Activating the NRF2 pathway

Since the Kelch-like ECH-associated protein 1 (KEAP1)-NRF2 pathway is the master regulator of the antioxidant response, activating NRF2 signaling can improve the overall cellular antioxidant capacity. It is not surprising that NRF2 is involved in various ocular diseases, such as glaucoma, retinopathy, cataracts, AMD and so on [179]. NRF2-deficient mice developed AMD-like symptoms featured by degeneration of RPE, accumulation of inflammatory markers and deregulation of autophagy [180]. Overexpression of NRF2 in the *Pde6b* mouse model sufficiently rescued the RPE and relieved the disruption in the cone photoreceptor layer. At the same time, transcriptome profiling showed that NRF2 upregulated multiple oxidative defense pathways, especially glutathione synthesis [181].

Sulforaphane, a major isothiocyanate derived from cruciferous vegetables, has shown its therapeutic effect on retinal degeneration models via the activation of NRF2, resulting in the increased level of TXN, TXNRD, and heme oxygenase-1 (HO-1) [103,182]. A new group of synthetic compounds exhibiting more potent NRF2 activation capacity have been tested in different ocular diseases models. A triterpenoid compound, RS9, was control-released in the vitreous body and maintained at 1 nM for 2 weeks in a rabbit RP model. The treatment significantly increased the NRF2 transcriptional activity and prevented the thinning of the ONL [183].

Most of the chemical NRF2 activators are electrophiles targeting the cysteine-rich domain of KEAP1 where NRF2 binds to and forms covalent adducts with the thiols by Michael addition reactions. If the concentration of the chemicals increases, it is unavoidable to cause the off-target effect that other cysteines involved in antioxidant defense get affected. The therapeutic window of these compounds is narrow and potential adverse effects may arise from this issue. Therefore, activating NRF2 by interfering with the protein-protein-interaction (PPI) between KEAP1 and NRF2 becomes an attractive alternative idea to overcome this later issue and find the more specific and safer NRF2 activators. With the help of co-crystal structures, more chemical scaffolds have been discovered via high throughput screening [184]. However, due to chemical properties of this class of molecules, there might be challenges for their pharmacokinetics and bioavailability.

Cepko's group tried to use AAV to express NRF2 protein in the RP mouse model. They found that compared to delivering antioxidant enzymes regulated by NRF2, such as SOD and CAT, greater cone survival was seen in retinal degeneration mice treated with AAV-NRF2 under the control of a CMV promoter. The gene therapy by AAV-NRF2 maintained retinal morphology, inhibited oxidative stress, and improved the function of retina. The treatment not only protected photoreceptors but also preserved the RGCs in the RP mouse model [185]. Thus, AAV-NRF2 may be an effective treatment of RP by providing antioxidant defense for multiple cell types in the retina.

### 4.3 Antioxidant enzymes

As we mentioned above, oxidative stress is a common phenomenon in inherited retinal diseases despite gene mutations, so that delivering antioxidant enzymes is a promising therapy to mitigate the symptoms. With the help of virus-based gene therapy, it becomes technically feasible.

Transfecting RPE cells with adenovirus carrying the *CAT* gene, even a small subgroup of cells, protected all cells from H_2_O_2_ insult. Subretinal injection of the virus into mice resulted in a high CAT activity in RPE which even the neighboring photoreceptors benefited from the treatment and became more resistant to photo-oxidative stress induced by bright light [186].

When Campochiaro's group overexpressed SOD1 in *rd1* mice, they surprisingly found the paradoxical effect that the overexpression accelerated the loss of cone function. SODs are the enzymes to convert superoxide anion into H_2_O_2_, elevated production of H_2_O_2_ by SODs may contribute to the toxic effect. When SOD2 and CAT were overexpressed in *rd10* mice simultaneously, the oxidative stress in photoreceptors was significantly lowered [187]. A later study from the same group confirmed that to achieve the protective effect, SODs must be co-expressed with an H_2_O_2_-detoxifying enzyme in the same cellular compartment [188].

Although no mutation of TXN family proteins has been reported in inherited retinal degeneration, attempts have been made to deliver TXN proteins as a therapeutic. Human RPE cells transfected with AAV-carrying *TXN2* became more resistant to lipid peroxides and apoptosis inducers. In addition, AAV-*TXN2* alleviated ER stress by upregulating heat-shock proteins (HSPs) [189]. Miranda's group intraperitoneally injected TXN into the *rd1* mice and observed several beneficial effects. TXN treatment slow-downed photoreceptor death and mitigated inflammation by reducing retinal macro- and microgliosis. In addition, TXN treatment increased the amount of GSH concentration and further supported the maintenance of redox balance in the retina [190]. However, it should be noticed that the TXN used in Miranda's study was *E. coli* Txn, not mammalian TXN1. The key difference is that *E. coli* Txn only contains the active site cysteine pair while mammalian TXN1 contains several structural cysteines (3 in human TXN1 and 4 in mouse TXN1). The structural cysteines in mammalian TXN are also important for some of their functions, such as protein binding and posttranslational modification, thus using *E. coli* Txn excluded the potential effects of the structural cysteines of mammalian TXN1 in retina [11]. Overexpression TXN downstream proteins, for example, MSRs or PRDXs, also exhibited the therapeutic potential to treat retinal degeneration [130,136,144].

### 4.4 Trick or treat, rescue photoreceptors via glucose metabolism, the example of rod-derived cone viability factor (RdCVF) and TXNIP

Retina needs large amount of energy to maintain its function and glucose is considered as the preferred source of energy [191]. Interestingly, retina exhibited a high level of aerobic glycolysis (known as the Warburg effect) as a non-proliferating tissue. Photoreceptors are thought to contribute most of the glycolysis in retina as retina lacking photoreceptors showed more than 50% reduction in glycolysis [192]. Although the Warburg effect is less efficient in terms of generating energy, it provides more metabolic intermediates for biosynthesis. A remarkable fact about the mammalian retina is the outer segment of photoreceptors gets completely renewed every 10 days and the process favors glycolysis for anabolism [193,194]. In addition, glucose can be directed into pentose phosphate pathway (PPP) to generate NADPH, which supplies electrons for the TXN and GLRX antioxidant systems. Failure in glucose usage has been accused of exacerbating the progress of several retinal degenerative diseases [195]. Thus, modulating glucose metabolism emerges as a promising therapeutic to treat retinal degeneration.

**Fig. 5:**
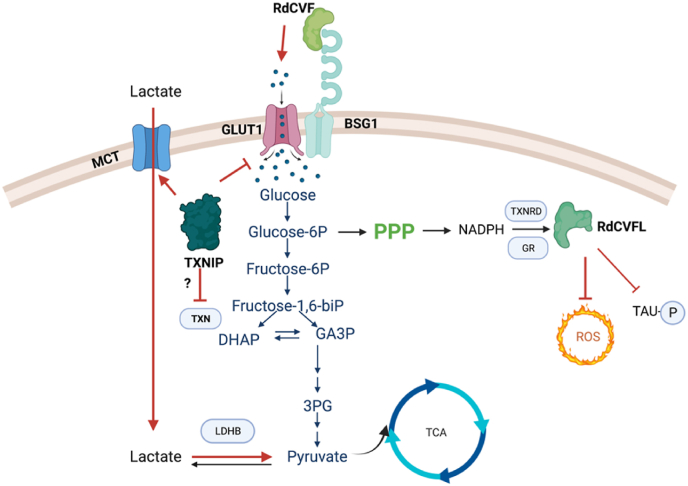
**RdCVF/RdCVFL and TXNIP as gene therapies for rescuing cones in retinal degeneration.** RdCVF binds to BSG1 and promotes the glucose uptake via GLUT1. Glucose can influx into PPP to generate more NADPH to support antioxidant enzymes. RdCVFL, as a TXN-like protein, participates in antioxidant defense and prevents the phosphorylation of TAU. TXNIP promotes the utilization of lactate as an alternative fuel, especially with the help of lactate dehydrogenase b (LDHB). However, it may bind to TXNs and interfere with their antioxidant functions.

Although diverse genetic mutations were found in RP, one phenomenon is shared in all patients: after the majority of rods die, cones start degenerating as a secondary event. The mystery was solved by the discovery of rod-derived cone viability factor (RdCVF), a protein serving as a trophic factor and promoting the survival of cones both *in vitro* and *in vivo* [196]. The protein is encoded by the *NXNL1* gene specifically expressed by photoreceptors. RdCVF is secreted by rods and interacts with basigin-1 (BSG1) that is bound to the glucose transporter GLUT1 on the membrane of cones. RdCVF binding promotes glucose uptake by cones and stimulates the aerobic glycolysis [197]. Interestingly, the *NXNL1* gene encodes another protein due to alternative splicing, RdCVFL (L for long), with a typical TXN-fold containing a catalytic cysteine pair at the active site. Mice lacking the *Nxnl1* gene are more sensitive to photooxidative damage. Viral delivery of RdCVF and RdCVFL protected photoreceptors from retinal degeneration caused by mutation and light [27,198]. RdCVFL, the TXN-like protein, showed the ability to inhibit TAU phosphorylation and support the activity of MSRA to reduce methionine sulfoxide [198,199], suggesting its potential role in antioxidant defense. Moreover, a recent study from our lab observed a prominent chaperone activity of RdCVFL and indicated its potential role in protein quality control in the retina [200]. The high competitive advantage of the *NXNL1* gene is to hit two birds, the redox and the metabolic modulation, with one stone [201] (Fig. 5).

Cepko's group found that systemic administration of insulin prolonged cone survival by stimulating the insulin/mTOR pathway in a mouse model of RP, highlighting the importance of glucose usage for photoreceptors [202]. Based on the concept, 20 genes related to glucose metabolism were tested in *rd1* mice, and only *TXNIP* showed beneficial effects. For this screening the transgene was under the control of a cone promoter (OPN1L/MW) and RdCVF was not active in those conditions as we reported previously [203]. Thioredoxin interacting protein (TXNIP) is an endogenous inhibitor of TXN which binds to reduced form of TXN but not the oxidized form [204]. The interaction between TXN and TXNIP is based on the disulfide formation between C^32^ in TXN and C^247^ in TXNIP [205]. Besides binding to TXN, TXNIP plays a negative-feedback role in glucose metabolism. TXNIP suppresses glucose uptake by binding to the glucose transporters (GLUTs) and reducing their mRNA level [206]. Phosphorylation and degradation of TXNIP can be triggered by the activation of cyclic AMP/protein kinase A (cAMP/PKA) signal transduction pathway during glucose uptake [207]. TXNIP was also reported to promote the utilization of non-glucose fuels in cells, such as lactate, which is imported by via monocarboxylate transporters (MTCs) [208,209]. AAV-*TXNIP* prolonged cone survival and visual acuity in *rd1* mice. The C247S mutant TXNIP, which loses its binding to TXN, showed a more robust rescue on cones, suggesting the interaction between TXN is a negative regulator of TXNIP. Interestingly, when lactate dehydrogenase b (LDHB), an enzyme that catalyzes the conversion of lactate to pyruvate, was co-delivered, the beneficial effect was amplified [210]. The current results suggest that TXNIP preserves cones in RP by enhancing their lactate utilization. However, the effect of delivery of TXNIP on the redox systems has not been totally clear yet. Arnér's group reported a surprisingly normal TXN system activity but a pronounced basal activation of NRF2 in myoblasts from the patients lacking TXNIP due to mutation [209]. The paradox between energy usage and redox regulation should be addressed in the future (Fig. 5).

## 5 Conclusions

Despite the genetic heterogeneity, oxidative stress has been considered as a hallmark of inherited retinal diseases contributing to the progress of retinal degeneration in many ways. Therefore, restoring the redox balance in retina can be a potential therapeutic strategy to slow down the deterioration. TXN and GLRX as the two major antioxidant systems in cells play versatile roles in retina. Recent findings of RdCVF/RdCVFL and TXNIP added more complexity into the system and provide another perspective on glucose metabolism. Although the mutations causing inherited retinal degeneration cannot be fixed, therapies modulating the antioxidant systems are greatly meaningful as preventive treatments and can buy more time for patients for future therapies.

## Author contributions

Conceptualization, X.R. and T.L.; writing—original draft preparation, X.R.; writing—review and editing, X.R. and T.L.; supervision, T.L.; funding acquisition, X.R. and T.L. All authors have read and agreed to the published version of the manuscript.

## Funding

X.R. was supported by grants from 10.13039/501100004047Karolinska Institutet (C25101033), the 10.13039/501100004359Swedish Research Council (C25101223), the 10.13039/100010810Eye Foundation in Sweden (Ögonfonden) in Sweden. T.L. was supported by 10.13039/501100001677Inserm.

## Declaration of competing interest

The authors declare the following financial interests/personal relationships which may be considered as potential competing interests: Dr. Thierry Léveillard holds a patent on the use of RdCVF and RdCVFL to treat retinal degenerations. No other conflicts of interest.

## Data Availability

No data was used for the research described in the article.
